# A 53-Year-Old Cyanotic Woman without Clubbing Fingers

**DOI:** 10.1186/1749-8090-10-S1-A38

**Published:** 2015-12-16

**Authors:** Cheng Hsin Lin

**Affiliations:** 1 Division of Cardiovascular Surgery, Department of Surgery, Wan Fang Hospital, College of Medicine, Taipei Medical University, Taipei, Taiwan

## Background/Introduction

Double-Chambered right ventricle (DCRV) is rare in patients with congenital heart disease, and even rare in the adults.

## Aims/Objectives

We report a case of subarterial ventricular septal defect (VSD) with DCRV in a 53-year-old lady presented with cyanosis and exercise intolerance without clubbing fingers. We also review the available reports in the literature for a concise summary of the rare condition.

## Method

A case report and literature review.

## Results

This patient had serial preoperative examinations including transthoracic and transesophageal echocardiographies, and cardiac catheterization before undergoing cardiac surgery for reconstruction of right ventricular outflow tract and repair of aortic and tricuspid valves. She had an uneventful postoperative course and remained asymptomatic afterward. Patients of similar conditions had been rarely reported with various presentations while the surgical results are excellent in most.

## Discussion/Conclusion

Double-Chambered right ventricle in the adult is a rare condition and can mislead the diagnosis into Eisenmenger Syndrome, while the two have extremely different prognosis. Our report demonstrates a successful surgical treatment of a patient with VSD and DCRV. Review of literature would raise the caution of clinicians about the diagnosis and pathophysiology of this condition.

## Consent

Written informed consent was obtained from the patient for publication of this abstract and any accompanying images. A copy of the written consent is available for review by the Editor of this journal.

